# Evaluation of circulating cell free DNA in plasma as a biomarker of different thyroid diseases^☆^

**DOI:** 10.1016/j.bjorl.2018.12.008

**Published:** 2019-02-18

**Authors:** Ozge Caglar, Begum Cilgin, Mustafa Eroglu, Akin Cayir

**Affiliations:** a Canakkale Onsekiz Mart University, Faculty of Medicine, Department of Head and Neck Surgery, Canakkale, Turkey; b Canakkale Onsekiz Mart University, Institute of Health Sciences, Canakkale, Turkey; c Canakkale 18 March University Hospital, Endocrin Department, Canakkale, Turkey; d Canakkale Onsekiz Mart University, Vocational School of Health Services, Canakkale, Turkey; e Human Nutrition Research Centre, Institute of Cellular Medicine, Newcastle University, Newcastle-upon-Tyne, United Kingdom

**Keywords:** Thyroid diseases, Cell free DNA, Plasma, Biomarker, Doenças da tireoide, DNA livre de células, Plasma, Biomarcador

## Abstract

**Introduction:**

Many studies have been done on proteomics, genomics, epigenetic, immunogenetics in many body fluids. Among these, circulating cell-free DNA (ccfDNA) entered the literature in 1948, but it has not been studied for many years due to technological deficiencies. Following recent advances, geno-metastasis has been mentioned and new research is needed in this area. ccfDNA is known to be an important biomolecule in this regard.

**Objective:**

The presence of cell-free DNA in the circulatory system may offer a tremendous opportunity to provide novel biomarkers for thyroid diseases. This experimental study was conducted to determine the amount of ccfDNA in different thyroid diseases, then to evaluate whether the ccfDNA concentration varied between the disease groups and control group.

**Methods:**

In total, we included 121 individuals in the present study. We collected blood samples and then determined the ccfDNA concentration in plasma of collected blood samples from three groups: thyroiditis (*n* = 33), benign (*n* = 37), and malignant (*n* = 30) and from a control group (*n* = 21).

**Results:**

The median values of the ccfDNA groups were found as 1610, 1665, 1685 and 576 ng/mL for the thyroiditis, benign, malign, and control groups, respectively. Findings showed that the ccfDNA of the three groups was significantly higher than the control (*p* < 0.0001). Each group was compared in terms of ccfDNA and the *p*-values of benign-thyroiditis, benign-malign, and thyroiditis-malign were 0.09, 0.65, and 0.29, respectively.

**Conclusions:**

The clear differences between thyroid diseases and controls suggest that ccfDNA is worthy of attention as a biomarker for further evaluation of different thyroid diseases. Likewise, it might indicate a clear tendency that ccfDNA can also be used to distinguish different thyroid diseases.

## Resumo

**Introdução:**

Muitos estudos foram realizados em proteômica, genômica, epigenética e imunogenética em vários fluidos corporais. Entre esses, o DNA circulante livre de células (cfDNA) despontou na literatura em 1948, mas não foi estudado por muitos anos devido a deficiências tecnológicas. Após recentes avanços, a genometástase é mencionada e novas pesquisas tornam-se necessárias nessa área. Nesse sentido, o cfDNA é conhecido por ser uma importante biomolécula.

**Objetivo:**

A presença de DNA livre de células no sistema circulatório pode oferecer uma excelente oportunidade para fornecer novos biomarcadores para doenças da tireoide. Este estudo experimental foi conduzido para determinar a quantidade de cfDNA em diferentes doenças da tireoide e então avaliar se a concentração de cfDNA variou entre os grupos com doença e o grupo controle.

**Método:**

No total, 121 indivíduos foram incluídos no estudo. Coletamos amostras de sangue e, em então, determinamos a concentração de cfDNA no plasma de amostras de sangue de três grupos: tireoidite (n = 33), benigno (n = 37) e maligno (n = 30) e de um grupo controle (n = 21).

**Resultados:**

As medianas dos valores dos grupos de cfDNA foram de 1.610, 1.665, 1.685 e 576 ng/mL para os grupos tireoidite, benigno, maligno e controle, respectivamente. Os achados mostraram que o cfDNA dos três grupos com doença era significativamente maior do que o do grupo controle (*p* < 0,0001). Cada grupo foi comparado em termos de cfDNA e os p-valores de benigno-tireoidite, benigno-maligno e tireoidite-maligno foram de 0,09, 0,65 e 0,29, respectivamente.

**Conclusões:**

Como resultado, as óbvias diferenças entre as doenças da tireoide e os controles sugerem que o cfDNA é digno de atenção como um biomarcador para avaliação adicional das diferentes doenças da tireoide. Da mesma forma, isso pode indicar uma clara tendência de que o cfDNA também pode ser utilizado para distinção das diferentes doenças da tireoide.

## Introduction

It has been estimated that 6.71% of the European population and 4.78% of the United States (U.S.) population with thyroid disorders has not been diagnosed,^1^ which represents half the number of people with the disease.^2^ A study performed in the U.S. showed that 4.6% and 1.3% of individuals were diagnosed with hypothyroidism and hyperthyroidism, respectively, in the screening of 13,344 individuals with previously-unrecognized thyroid disease.^3^ Similar findings observed in a meta-analysis study showed that 4.94% and 1.72% of individuals were diagnosed with hypothyroidism and hyperthyroidism respectively.^4^

Thyroid dysfunctions and hypothyroidism have become more common in recent years in the middle age group and in women as a result of increased thyroid inflammation. Thyroiditis is evaluated in three categories according to its healing time: acute thyroiditis (fast onset and short duration), delayed thyroiditis (lasting less than a year), and chronic thyroiditis (life-long). Among these, Hashimato thyroiditis is a chronic thyroid pathology that is the most common life-threatening inflammation of the thyroid. Although the cause is not completely known, some factors including use of iodized salts, infections, and exposure to radiation are considered to be effective in the formation of Hashimoto's thyroiditis.

Among thyroid diseases, 3–7% of people with thyroid nodules has a thyroid disorder. According to research, most of these nodules are benign tumors and 5–8% of them can be converted to malignant tumors^5^ which are the most common tumors of the endocrine system. The clinical significance of thyroid nodules is based on the need to exclude thyroid cancer, which is seen in 7–15% of cases, depending on age, gender, radiation exposure history, family history, and other factors.^6^, ^7^ There are four major types of cancer in the thyroid gland. These are, in order of frequency: papillary, follicular, medullary, and anaplastic carcinomas. A considerable number of cancers are follicular cells.^5^ Differentiated Thyroid Cancer (DTC) including papillary and follicular carcinoma constitutes the majority (>90%) of all thyroid cancers.^8^

Currently, Fine Needle Aspiration Biopsy (FNAB) is used to diagnose and treat malignant tumors, but a 20% failure rate occurs due to the lack of adequate specimen and specific tissue, cytologic techniques and different methods used by technicians. Therefore, there is a need for new, more reliable biomarkers able to diagnose thyroid cancer early and definitively, which can be measured in a non-invasive, easy and accurate manner. For this purpose, many studies have been carried out on proteomics, genomics, epigenetics, immunogenetics and other molecules in various bodily fluids. Among these, circulating cell-free DNA (ccfDNA) entered the literature in 1948 but has not been studied for many years due to technological inadequacies. Following recent technological developments, the mechanism of metastasis has begun to be discussed again, and mention of geno-metastasis, leading to the need for new research in this area. ccfDNA is an important bimolecular in this regard. This molecule gains attention in many diseases, mainly in cancer studies at the genomic and epigenomic level, in relation to cancer type, development, amount of blood plasma, and other molecules in the plasma. For example, such data is very promising in determining whether or not a person has cancer and if the cancer has metastasized in the body, whether success has been achieved following treatment, and whether surgery is necessary.

In the present study, we aimed to measure the amount of ccfDNA in plasma samples collected from individuals with thyroid diseases and healthy individuals, and then to compare the amount of each groups. The realization of this study will make an important contribution toward determining whether the level of ccfDNA can be used as a molecular biomarker for different thyroid diseases.

## Methods

The present study was conducted on the blood samples of 100 thyroid patients. Of the 100 patients included in our study, 33 were patients with thyroid disease, 37 patients had a benign thyroid nodule, and 30 patients had thyroid cancer. For the control group, 21 patients with no known additional diseases were included. We performed additional examination of patients with nodules or masses in the thyroid. Patients were diagnosed with thyroid USG, followed by FNAB, however, some cancer patients were definitively diagnosed after undergoing an operation. Only the patients who had thyroid disease were evaluated in the present study. Before taking peripheral blood samples, the patients and controls were informed about the study and its aims and then each gave their consent by signing the appropriate form. The characteristics of the patients and the controls were collected by means of a questionnaire. Ethical permission was granted by the Faculty of Medicine Ethics Committee (27/2016-E.70096).

For the ccfDNA, 5 mL blood was collected in sterilized tubes containing K3-EDTA. After taking the blood samples, they were immediately centrifuged at 3000 g for 10 min. The plasma samples were transferred to another sterile, DNAse-free tube and the samples were centrifuged at 16,000 g for 10 min. Then, the plasma samples were separated and stored at −80 °C for further study. The DNA content of the plasma samples was measured directly with a fluorescence-based Quant-iT™ high-sensitivity DNA assay kit and a Qubit® fluorometer (Invitrogen, Carlsbad, CA, USA). The Qubit® 2.0 Fluorometer is used for quantitation of DNA, RNA, and protein. It uses specific dyes for each type of molecule which have extremely low fluorescence until they bind to their targets. After binding, they give off an intensely fluorescent signal which is directly proportional to the DNA concentration of any solution. In the present study, the ccfDNA of each individual was measured using a DNA curve obtained from the DNA standard of known concentrations. Plasma samples were analyzed in duplicate and the mean of the two values was used as the final DNA amount. The between-measurements and coefficients of variation of this assay were less than 1.00%.

Differences between groups were detected using one-way ANOVA then Bonferroni as a post hoc test. The differences between male and females were analyzed using Mann–Whitney *U* test. Significance levels were indicated. The effects of confounding factors (age, gender, smoking, habits, etc.) were also evaluated statistically. The contribution of each factor taken into consideration was evaluated using multiple regression analysis. Possible associations were investigated with Pearson correlation. SPSS 18.0 program was used for statistical calculations.

## Results

The age and gender distribution of each group are presented in Table 1. The majority of patients in our study were female. Thirty (90.9%) had thyroiditis and 64.9 (24%) had benign nodules. Twenty-three (76.7%) of the malignant patients were females. The mean age was 37.6 for those with thyroid diseases, 54.1 in benign nodules, and 47.8 for cancer patients.

**Table 1:** Characteristics of the populations.

	Control (*n* = 21)	Thyroiditis (*n* = 33)	Benign (*n* = 37)	Malign (*n* = 30)
Age	30.1 ± 15.2	37.6 ± 10.9	54.1 ± 13.1	47.8 ± 11.9
Male	29.8 ± 15.8 (43%)	47.3 ± 7.5 (10%)	58.9 ± 10.1 (33%)	46.1 ± 13.4 (23%)
Female	30.4 ± 15.4 (47%)	36.7 ± 10.8 (90%)	51.7 ± 13.9 (67%)	48.3 ± 11.8 (77%)

In the case of smoking and alcohol use, 8 (23.5%) of the thyroid patients were active users, 6 (17.8%) were former users, and 7 (20.5%) reported active alcohol use. Among the malignant patients, 13 (43.3%) patients were active users, 3 (10%) had used alcohol before, and 7 (23.3%) patients used alcohol currently. In the benign nodule patients, 6 (15.7%) patients reported active cigarette use, 6 (15.7%) patients had the habit previously, and 4 (10.5%) patients reported continuing alcohol use. The control group consisted of 9 (42.85%) males and 12 (57.14%) females. The mean age was 30.1 in the control group.

**Figure 1:**
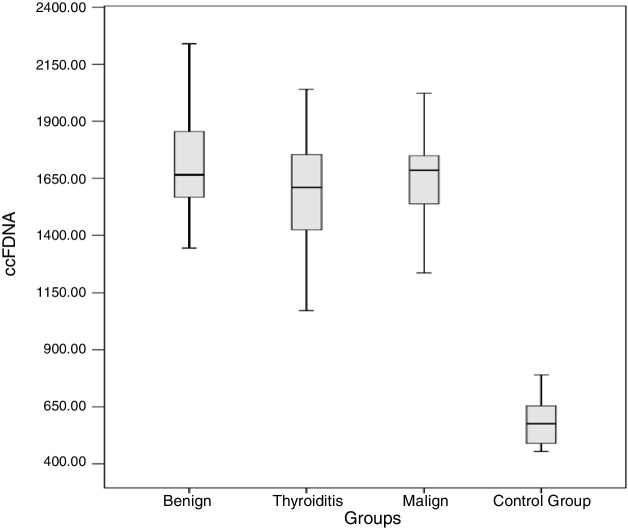
ccfDNA amount in each group.

The results obtained for ccfDNA in each group are presented in Table 2 and Fig. 1. According to the results obtained, the minimum and maximum values of ccfDNA in the patients were found to be 908–2280 ng/mL for thyroiditis, 1344–2560 ng/mL for benign, 1212–2840 ng/mL for malign, and 454–1018 ng/mL for the controls. The median values of the ccfDNA groups were found as 1610, 1665, 1685, and 576 ng/mL for the thyroiditis, benign, malign, and control groups, respectively.

**Table 2:** ccfDNA in diseased and control groups.

ccfDNA	Control (*n* = 21)	Thyroiditis (*n* = 33)	Benign (*n* = 37)	Malign (*n* = 30)
Total mean	623.3 ± 172.9	1613 ± 284	1721.3 ± 243	1691 ± 292
Minimum	454	908	1344	1212
Maximum	1081	2280	2560	2840
Male	608.0 ± 120	1667 ± 217	1825 ± 310	1687 ± 251
Female	634.8 ± 208	1608 ± 292	1664 ± 181	1692 ± 309

Four groups including control, malign, benign, and thyroiditis were compared using one-way ANOVA then Bonferroni as a post hoc test. According to the obtained results, ccfDNA in the three patient groups was significantly higher than the controls (*p* < 0.0001 for all groups). Each group was compared in terms of ccfDNA and the *p*-values of benign-thyroiditis, benign-malign, and thyroiditis-malign were 0.09, 0.65, and 0.29, respectively.

The ccfDNA of males with thyroiditis was slightly higher than the females; however, this difference did not reach a significant level (*p* = 0.7). The smoker thyroiditis patients (1658 ng/mL) had higher ccfDNA when compared with non-smoker patients (1599 ng/mL). However, the difference was not statistically significant (*p* > 0.05). Analysis revealed no correlation between ccfDNA and age. Linear regression analysis showed that ccfDNA was not affected by confounding factors such as age, gender, smoking, and the alcohol habit (*p* > 0.05).

In this group, there is a clear significant tendency toward males (1825 ng/mL) than in females (1664 ng/mL) in terms of ccfDNA (Mann Whitney U, *p* = 0.054). The amount of ccfDNA in smokers and nonsmokers is similar and thus there is no relationship between smoking and ccfDNA. It was observed that the ccfDNA in benign individuals did not correlate with age (*p* > 0.05). Linear regression analysis indicated that gender might affect ccfDNA in benign patients in the model adjusted by age, gender, smoking, and alcohol habit (*p* = 0.08).

In terms of gender, there is no difference between males and females. Similarly, it was observed that the ccfDNA of smokers and non-smokers is similar. In the malign nodule group we did not observe any correlation between ccfDNA and age. In the regression analysis, no potential factors were determined which affected the amount of ccfDNA in the plasma samples.

As a group containing three different diseases, the ccfDNA of males (1762 ng/mL) was higher than females (1651 ng/mL), which had a tendency to be significant (*p* = 0.08). In terms of the smoking habit, the amount for smokers and non-smokers’ ccfDNA was very close (*p* > 0.05). According to Spearman correlation, the ccfDNA is positively correlated with age at a significant level (*r* = 0.21, *p* < 0.05). In multivariate regression models adjusted by age, gender, smoking, and alcohol habit, the ccfDNA did not associate with any factors in the models. After backward method was applied to the same analysis, age was the sole factor that affected ccfDNA in thyroid disease.

## Discussion

In many diseases, especially for cancer, it is crucial to find surrogate samples due to the invasiveness of the samples. Today, it is well-known that ccfDNA can be measured in healthy individuals in body fluids including plasma, serum, sputum, and urine.^9^ On the other hand, ccfDNA can also be measured at higher levels in other illnesses including autoimmune diseases, several cancers and cardiovascular diseases.^9^, ^10^, ^11^ More specifically, the characterization of ccfDNA in respect of its mutations, epigenetic alterations, and fragment size and distribution have been associated with several types of cancer.^12^ Thus, non-invasive biomarkers enable us to follow the diagnosis, prognosis and monitoring of diseases.

Although the origin of ccfDNA is not clear, there are several mechanisms that contribute to the amount of circulating DNA. The mechanisms contributing to ccfDNA may change depending on the type and/or stage of the disease. It has been accepted that dead or dying cells are the main source of ccfDNA. However, it has also been proven that living cells can release ccfDNA. Furthermore, the amount in circulation may vary depending on the disease stage. Above all, cell breakdown, apoptosis, necrosis, exosome, and virtosomes are accepted as the main mechanisms. In a suggested model, it is supposed that there are three events responsible for the ccfDNA in the blood of a cancer patient, including necroptosis, apoptosis, and secretion by tumor and macrophages.^13^ Due to these events, ccfDNA can be released single-stranded or double-stranded, and depending on the tumor type, different forms of ccfDNA may be released.^13^

In the present study, we determined that the ccfDNA measured in groups having three different types of thyroid disease was consistently significantly higher than the control group. We found that the ccfDNA in thyroiditis, benign, and malign groups were 2.59, 2.76, and 2.72 times higher than the control group. Therefore, we present ccfDNA amount in plasma samples as a novel biomarker of thyroid diseases. Among the thyroid diseases, there were no clear differences. However, the higher ccfDNA of benign compared with thyroiditis might indicate that ccfDNA can be used to show the difference between thyroid diseases.

The rationale behind thyroid cancer is not yet fully understood. However, it has been suggested that there are multiple factors including radiation, environmental pollution, and nutrition that cause the individual to be prone to thyroid cancer. All multifactorial causes have the ability to induce apoptotic mechanisms.^14^ As indicated above, environmental exposure can affect the mechanisms associated with the ccfDNA amount.^15^ The mechanisms regarding ccfDNA release are also associated with thyroid diseases. For example, it has been stated that apoptosis is associated with homoeostasis of the thyroid gland. It also plays a role in the mechanisms of autoimmune thyroiditis and in thyroid câncer.^16^ In this regard, the higher quantity of ccfDNA in the present study can be attributed to apoptotic mechanisms. In another study, there was a significant difference in the ccfDNA integrity index between patients with benign nodules and patients with cytologically thyroid carcinoma. This finding supports ccfDNA as a promising biomarker for the diagnosis of thyroid nodules.^17^ In our study, we included these patients in addition to follow-up patients with the cause of thyroid disease (thyroiditis).

The abnormal increase in the amount of ccfDNA in serum and plasma in cancer patients was detected for the first time in 1977.^18^ However, the use of prognosis and diagnosis as a marker has only recently been addressed.^19^ In a large group of cancers including colorectal, pancreas, lung, bladder, bass-neck, and liver cancer, free DNA mutations were detected in the ccfDNA.^20^, ^21^, ^22^, ^23^, ^24^, ^25^ Several DNA alterations have been reported in free DNA, including point mutations, DNA hypermethylation, Microsatellite Instability (MI), and Heterozygous Loss (LOH) (A). In many cases, these changes are the same as those found in the patient's primary tumor site and support the tumoral origin of the altered free DNA. Changes in ccfDNA are not confined to any particular tumor region, species or grade. However, patients with late stage disease and metastasis tend to have significantly higher levels of ccfDNA. Thus, in the present study, ccfDNA can also provide a valuable resource of genetic material as a surrogate for molecular analysis in thyroid diseases.

It has been well known that larger data could provide more precise results in such a study. The small number of participants is a limiting aspect of our study. Despite this situation, the statistically significant differences between the diseases groups and the controls supported the ccfDNA as a distinct feature for thyroid diseases which could be used for diagnosis, prognosis and monitoring of diseases.

## Conclusion

The differences between thyroid diseases and the control group suggest that cfDNA should be noted as a biomarker for further evaluation of thyroid diseases. In addition, we offer several suggestions. First, it is important to develop quantitative approaches to determining the burden of DNA changes in ccfDNA and to establish a lower detection limit for the methodologies used. This will be crucial for determining the cutoff values for the significance of the determination of DNA changes. Second, future work should consider incorporating different genes and modification types to increase sensitivity and specificity for cancer detection. Third, systematic comparisons between different cancers should be made to determine whether some types of cancer are more prone to releasing modified ccfDNA than others. Fourth, it is very important to develop large-scale studies based on case-control and/or prospective study designs instead of analyzing sequentially-recruited clinical cases. Fifth, the transformation of experimental ccfDNA studies into epidemiological and clinical practice requires the development of instrumentation and methods adapted to large-scale, low-cost analysis of mutations in DNA in very small quantities. Given the potential for cancer management of ccfDNA, these developments may quickly lead to important applications in cancer detection, diagnosis, prognosis, and follow-up treatment.

## Funding

This work was supported by **Çanakkale Onsekiz Mart University** The Scientific Research Coordination Unit ((BAP), Project number: TYL-2017-1114).

## Conflicts of interest

The authors declare no conflicts of interest.
